# Factors associated with cervical cancer screening uptake among Inuit women in Nunavik, Quebec, Canada

**DOI:** 10.1186/1471-2458-13-438

**Published:** 2013-05-03

**Authors:** Helen Cerigo, Francois Coutlée, Eduardo L Franco, Paul Brassard

**Affiliations:** 1 Centre for Clinical Epidemiology, Jewish General Hospital, Montreal, Canada; 2 Department of Epidemiology, Biostatistics and Occupational Health, McGill University, Montreal, Canada; 3 Department of Oncology, McGill University, Montreal, Canada; 4 Department of Medicine, McGill University, Montreal, Canada; 5 Department of Microbiology, Centre Hospitalier de l’Université de Montréal, Montreal, Canada

## Abstract

**Background:**

The Canadian circumpolar Inuit population has a higher incidence rate of cervical cancer than the general population and the majority of cases occur among underscreened women. The objectives of this study were to determine Pap smear utilization rates and to determine factors associated with time-inappropriate use of cervical cancer screening among a cohort of Inuit women from Nunavik, Quebec, Canada.

**Methods:**

This study utilizes baseline information collected from a cohort formed between January 2002 and December 2007 to study the natural history of HPV among Inuit women aged 21–69 years in Nunavik, Quebec. Cervical cancer screening history and other variables were obtained from a baseline questionnaire and medical chart review. Unconditional logistic regression was used to estimate the odds ratios and 95% confidence intervals for potential predictors of not having a Pap smear within the previous 3 years prior to cohort entry.

**Results:**

A total of 403 Inuit women who had a baseline questionnaire and chart review were included. The mean age of the study population was 34.2 years. In the three years prior to study entry, 25% of women did not have a Pap smear. Older age and never giving birth were significant predictors of time-inappropriate Pap smear use.

**Conclusions:**

Our results suggest that older women and women who are not accessing reproductive care have a lower compliance with time-appropriate cervical cancer screening and future research should address potential strategies to increase screening coverage among this group.

## Background

Early detection of cervical cancer and pre-cancer by the Papanicolaou (Pap) smear has led to the successful reduction of cervical cancer incidence and mortality throughout Canada. However, underutilization of cervical cancer screening is still a major problem, as 60% of cervical cancers occur in women who have not been screened in the three previous years [1,2]. In Canada, a variety of factors have been shown to predict underutilization of cervical cancer screening, such as older age, single marital status, lower educational attainment, birth place outside Canada, Aboriginal identity, rural residence, obesity and not having a family doctor [3-6]. Additionally, the few studies that have looked at reproductive and sexual behaviour factors found that ever being pregnant, early age at first sexual intercourse, use of birth control, number of sexual partners, and history of sexually transmitted infection (STIs) were associated with Pap smear use, although not consistently [7,8].

Among the Canadian Inuit population, age-standardized cervical cancer incidence rates are 2.5 to 3 times higher than the national average [9,10]. Inuit populations also face a higher cervical cancer mortality rate and a high prevalence of human papillomavirus (HPV), a necessary cause of cervical cancer [10-13]. About one-fifth of Canada’s Inuit population resides in Nunavik, the arctic and subarctic region of northern Quebec [14]. In Quebec, there is no organized cervical cancer screening program, and thus in Nunavik, all Pap smear screening is done opportunistically. The Pap test is available in all 14 communities of Nunavik and is generally performed by nurse practitioners. As women visit the community clinic for any reason staff will review their charts and offer cervical cancer screening if there is no history of Pap smear within the past year. Screening is also promoted and offered during prenatal and well-baby clinics. It is recommended that women get screened in the month of their birth, but there is no systematic follow-up or reminder system. Additionally, in association with a study on the natural history of HPV among Inuit women, members of the research team conducted health promotion activities around cervical cancer screening on the local radio three times per year during study recruitment and follow-up. Screening and treatment of cervical cancer is available at no charge under the provincial health insurance plan (Régie de l’assurance maladie du Québec).

A recent meta-analysis on the relative risk of cervical cancer among indigenous women of Australia, Canada, New Zealand and the United States found that although indigenous women had a high risk of invasive cervical cancer and mortality from this disease, they were not at an elevated risk of early-stage cervical cancer compared to the general population [15]. The authors suggest that this is likely the result of barriers to screening and adequate treatment after diagnosis. Therefore, understanding the predictors of inappropriate screening use is important, as groups of women who have lower compliance to screening guidelines represent potential targets for future screening interventions. Increasing their compliance will be essential to reducing cervical cancer incidence and mortality in Nunavik. The only known study that has examined the determinants of Pap smear utilization among Inuit women in Nunavik found that higher education levels and younger age were associated with screening within the previous two years [16]. Pap smear history was obtained through self-report in that study, which may introduce a recall bias because it has been shown that women consistently over-report their screening history [17,18]. Alternative methods to ascertain screening history, such as medical record review or linkage to screening registries would be preferable to avoid measurement error. The purpose of this report was to determine Pap smear utilization rates from medical chart review and to determine factors associated with time-inappropriate cervical cancer screening among a cohort of Inuit women from Nunavik, Quebec.

## Methods

This analysis utilizes baseline questionnaire and retrospective chart review data collected on a cohort of Inuit women, which was created to examine the natural history of HPV in Nunavik [12,19,20]. Briefly, between January 2002 and December 2007, a total of 621 Inuit women between the ages of 15 and 69, who had an intact uterus and no diagnostic suspicion for cervical cancer were recruited to this cohort, when presenting for a Pap smear (scheduled or otherwise). Full details on cohort eligibility and data collection are available elsewhere [12]. Written informed consent was obtained from all study participants and ethical approval for this study was obtained from the McGill Institutional Review Board.

To be eligible for this sub-analysis women had to have both a completed questionnaire and full medical chart review, and be between 21 and 69 years of age. Women under 21 years were excluded because they fall outside of the recommended age to initiate cervical cancer screening in Canada and in Quebec [21]. A three-year screening interval commencing at age 18 prevailed as the recommended screening procedure during the study period in Quebec and Canada [1,21].

Sociodemographic, reproductive, medical, sexual history and lifestyle characteristics for participants were obtained from a questionnaire administered at cohort entry and a baseline medical chart review. During the medical file review, a detailed history of cytology use was collected and this information was used to develop a binary outcome variable that distinguishes between women who had their last Pap test within the three years prior to cohort entry (time-appropriate) and those who did not have a Pap test within the three previous years, including those who never had a Pap test (time-inappropriate). The candidate predictor variables analysed in this study were age, marital status, employment status, education level, smoking status, alcohol use, self-reported history of STI, age at first sexual intercourse, number of lifetime sexual partners, number of lifetime deliveries, use of any birth control, and community of residence.

Descriptive statistics were obtained and chi-square tests (categorical data) and t-tests (continuous data) were used to assess associations between Pap testing and covariates. Relationships between all covariates were explored with cross-tabulations of categorical variables and correlation matrices of continuous variables to examine the presence of collinearity and interaction [22]. Unconditional logistic regression was used to examine the potential associations between all covariates and time-inappropriate cervical cancer screening. The following variables were selected for inclusion in the multivariate model based on *a priori* evidence of association in different populations: age, marital status and education [3,4]. Additional variables were considered for inclusion in the multivariate models if they were significantly associated with time inappropriate Pap smear use in an age-adjusted analysis (data not shown). Lifetime deliveries and current use of birth control were found to exhibit a high degree of collinearity, and thus only the lifetime deliveries covariate was included in the final model. Odds ratios (OR) and 95% confidence intervals (95% CIs) were calculated, adjusted for all variables in the model. Data management and statistical analysis were performed with SAS version 9.2 and statistical significance for all regressions was set at 5%.

## Results

Of the 621 cohort participants, 510 (82.1%) women had a complete questionnaire and full medical file review with cytological history*.* Among these women, 403 (79%) were between the ages of 21 and 69 and were therefore included in this analysis*.* All eligible women were sexually active prior to cohort entry. Selected demographic characteristics are displayed in Table 1. The average age was 34.2 years, with a range of 21 to 65 years. A total of 96 women (24.8%) had not had a Pap smear within the three years prior to cohort entry. Among those who did not receive a time-appropriate screening, 16 (16.7%) had never received a Pap smear. The highest rate of time inappropriate Pap smear use by age group was seen among women aged 45 and older (29.8%), followed by women aged 30 to 44 years (27.5%). There was a lower rate of time inappropriate cervical cancer screening among women 21–29 years (17.9%).

**Table 1:** Demographic characteristics of all study participants and by history of Pap smear within the previous three years (n=403)

	**All women (n = 403 )**	**Women screened in previous 3 years (n = 307) n(%)**	**Women not screened in previous 3 years (n = 96) n(%)**
Age Category^**†**^			
21-29	168	138 (82.1)	30 (17.9)
30-44	178	129 (72.5)	49 (27.5)
45+	57	40 (70.2)	17 (29.8)
Marital status			
Married or living with partner	253	190 (75.1)	63 (24.9)
Single	144	112 (77.8)	32 (22.2)
Missing	6	5 (83.3)	1 (16.7)
Educational attainment			
< Grade 9	126	91 (72.2)	35 (27.8)
≥ Grade 9	266	209 (78.6)	57 (21.4)
Missing	11	7 (63.6)	4 (36.4)
Employed			
Yes	306	226 (73.9)	80 (26.1)
No	89	73 (82.0)	16 (18.0)
Missing	8	8 (100.0)	0 (0.0)
Current smoker			
Yes	295	226 (76.6)	69 (23.4)
No	101	77 (76.2)	24 (23.8)
Missing	7	4 (57.1)	3 (42.9)
Current alcohol use			
Yes	303	232 (76.6)	71 (23.4)
No	95	71 (74.7)	24 (25.3)
Missing	5	4 (80.0)	1 (20.0)
Self-reported history of STI			
Yes	302	234 (77.5)	68 (22.5)
No	86	60 (69.8)	26 (30.2)
Missing	15	13 (86.7)	2 (13.3)
Lifetime no. of sexual partners			
Less than 10	220	166 (75.5)	54 (24.5)
10 or more	151	116 (76.8)	35 (23.2)
Missing	32	25 (78.1)	7 (21.9)
Age at first sexual intercourse^**††**^			
< 16 years	196	156 (79.6)	40 (20.4)
≥16 years	182	131 (72.0)	51 (28.0)
Missing	25	20 (80.0	5 (20.0)
Previously given birth			
Yes	364	281 (77.2)	83 (22.8)
No	38	25 (65.8)	13 (34.2)
Missing	1	1 (100.0)	0 (0.0)
Current use of birth control			
Yes	167	138 (82.6)	29 (17.4)
No	221	157 (71.0)	64 (29.0)
Missing	15	12 (80.0)	3 (20.0)
Community			
1	212	157 (74.1)	55 (25.9)
2	49	43 (87.8)	6 (12.2)
3	96	71 (74.0)	25 (26.0)
4	44	34 (77.3)	10 (22.7)
Missing	2	2 (100.0)	0 (0.0)

^+^ Mean (SD): 34.19 (9.56), Median: 32.12, Range: 21–65.

^++^ Mean (SD): 15.51 (2.23), Median: 15, Range: 10–30.

Table 2 shows unadjusted and adjusted odds ratio estimates for the association between candidate variables and time-inappropriate Pap smear use. In the fully-adjusted model, age was strongly associated with time-inappropriate Pap smear use. Women aged 30 to 44 had an over three times higher odds of having a time-inappropriate Pap smear than women 21 to 29 years (OR= 3.23, 95%CI: 1.72-6.04). Women 45 and older had a significantly higher odds of time-inappropriate Pap smear use in the univariate model (OR=1.99, 95%CI: 1.03-2.83), but this association did not maintain its significance in the multivariate model (OR=2.28, 95%CI: 0.99-5.26). No history of childbirth was also found to be a strong predictor of time-inappropriate Pap smear, where women without a history of childbirth had over a two times higher odds of having a time-inappropriate Pap smear than women with a history of childbirth (OR =2.57, 95%CI: 1.10-6.01). Community of residence had an effect on cervical cancer screening in the univariate analysis with community 1 having a significantly higher odds of time-inappropriate Pap smear use than community 2 (OR= 2.51, 95%CI: 1.01-6.22), but this association was not significant in the multivariate model.

**Table 2:** **Determinants of time-inappropriate Pap smear use**^**+ **^**among Inuit women in Nunavik, Quebec (n=403)**

		**OR (95% CI)**^**++**^
**Variable**	**Crude**	**Fully-adjusted**
Age	**1.38 (1.09 -1.74)**	
21-29	Reference	Reference
30-44	**2.63 (1.56-4.44)**	**3.23 (1.72-6.04)**
45+	**1.99 (1.03-3.83)**	2.28 (0.99-5.26)
Marital status at baseline		
Married or living with partner	Reference	Reference
Single	0.86 (0.53-1.40)	0.89 (0.49-1.62)
Educational attainment		
< Grade 9	Reference	Reference
≥ Grade 9	0.71 (0.44-1.16)	0.87 (0.47-1.59)
Employed		
No	Reference	
Yes	1.62 (0.88-2.94)	
Current smoker		
No	Reference	
Yes	0.98 (0.58-1.67)	
Current alcohol use		
No	Reference	
Yes	0.91 (0.53-1.54)	
Self-reported history of STI		
No	Reference	
Yes	0.67 (0.39-1.14)	
Age at first sexual intercourse (per year)	1.10 (0.98-1.21)	
Lifetime # of sexual partners		
< 10 partners	Reference	
≥ 10 partners	0.93 (0.57-1.51)	
Previously given birth		
Yes	Reference	Reference
No	1.76 (0.86-3.59)	**2.57 (1.10-6.01)**
Current use of any birth control		
No	Reference	
Yes	**0.52 (0.31-0.85)**	
Community		
1	**2.51 (1.01-6.22)**	1.82 (0.69-4.82)
2	Reference	Reference
3	2.52 (0.96-6.64)	1.95 (0.69-5.48)
4	2.11 (0.70-6.38)	1.16 (0.33-4.16)

^+^ No history of Pap smear within the three years prior to cohort entry.

^++^Odds ratio (95% Confidence Interval). Odds ratios are unadjusted or fully adjusted for all other variables in the model.

## Discussion

The analysis presented here refers to Pap smear use and determinants of time-inappropriate cervical cancer screening among Inuit women aged 21–69 in Nunavik, Quebec. Overall, there was a sizeable high-risk group who had not been screened in the three years prior to cohort entry (25%), and 4% of the sample had never been screened for cervical cancer. Although Aboriginal populations in Canada have traditionally had lower screening rates than the general population, we found that the cervical cancer screening coverage in this population was comparable to the screening coverage reported for the Canadian population [3,4,23]. However, there is still cause for concern, given that there is a high prevalence of high-risk HPV (HR-HPV) infection and incidence of cervical cancer in this population [9,10,12]. If high-risk subsets of the population are systematically underscreened for cervical cancer, then it is likely that incidence and mortality reductions will not be observed, despite screening rates that are comparable with the general population of Canada. Understanding, the predictors of screening underuse will help determine opportunities to adapt or enhance health promotion activities for screening among underserved groups.

In this population, older age and no history of childbirth were significant predictors of time-inappropriate Pap smear use. We found that older women had a significantly higher odds of not being screened appropriately compared to younger women. A similar effect of Pap smear rates decreasing with age was found in other Canadian populations [4,24]. It is worrisome that older women are at risk of not being screened appropriately, as cervical cancer mortality increases with age and cervical cancer incidence is higher among women 40 years and older [25]. Menopausal or post-menopausal women may not always realize that screening is still necessary after reproductive years [26], as younger women may be more aware of the need for periodic screening.

Our results also suggest that screening may be related to reproductive care use, given that women who had no history of child birth were more likely to have had a time-inappropriate Pap smear. In Nunavik, Pap smear is offered as part of the prenatal and well-baby visits, so women who access these services would likely have multiple opportunities to be screened. Similarly, women from Ontario without a prenatal visit during a three year interval were also less likely to have been screened for cervical cancer than those who did attend a prenatal visit [24]. Although we were unable to include use of birth control in the final model due to collinearity, in the univariate analysis we found that women who did not use birth control at baseline had an increased odds of having time-inappropriate screening. This is consistent with a previous study which found that women participated in screening because it was linked with the annual renewal of their oral contractive prescription [27]. Indeed, the majority of women in our study (78%) who used birth control used a hormonal birth control type (oral contraceptive or Depo-Provera injections), which might explain some of this relationship.

We were not able to replicate the finding that single or less educated women were less likely to have time-appropriate screening [4]. Although we were unable to investigate this finding further, differences in demographic and family structure characteristics of Nunavik, compared to the Canadian population in general may explain this null result [28,29].

Geographic variation in screening rates was found in the univariate analysis, with one community being highly predictive of time-inappropriate cervical cancer screening, but this association did not maintain significance in the multivariate analysis. Although this study was not able to uncover the factors that contribute to these variations, previous studies suggest that community size [7], access to female health providers [30], perceived importance of screening by health providers [31], and promotion of screening in the community [32] may play a role. The population size of each community is small (under 2500 people) and health centres are easily accessible by foot in all communities. In the univariate model women from the largest community were found to have a significantly lower odds of time-appropriate Pap smears, suggesting that community size may contribute to the variation by community. The two other communities also showed an elevated, but non-significant odds of time-inappropriate Pap smear use compared to the referent community in both the univariate and multivariate analysis. However, communities 2, 3 and 4 had similar population sizes and therefore size may not be the only factor contributing to the effect of community. Factors such as attitudes around screening in the community and characteristics of the health centre staff are likely to also contribute to these differences. It has been previously shown that Inuit women have a strong preference for having screening performed by a female provider [30]. Although, we were unable to assess the gender ratio of providers in these communities during data collection, the presence of a male provider may have led to a delay in screening acceptance among some women, which could help explain some of our findings. In the 2004 Nunavik Health Survey, 41% of women who had a Pap smear two or more years ago or never reported that cervical cancer screening was not offered by their doctors [16]. The factors related to the relationship between women and their health care providers are especially important because women can only access health care from their community’s health centre.

A limitation of this study was that women were recruited non-randomly, while attending any visit at the health centre that necessitated a Pap smear. Given that our sample was comprised of women who consented to the cohort study, it is likely that we underestimated the proportion of women who have never had a Pap test. It is difficult to assess the extent of this underestimation given that there is no cervical screening registry in Quebec; however, one randomly-selected population based study found that 6.8% of women aged 18–29 years across Nunavik were never screened [16], whereas a convenience-sample of women 18–63 years in two communities of Nunavik found that only 3.4% had not previously received a Pap test [30]. Our results are within the range reported among other samples in Nunavik, but caution must be exercised in comparison of these results due to the reported age-differences and the self-reported nature of cervical cancer screening history in these studies. Also, our sample was comparable to the general population of women in Nunavik, with the exception of an underrepresentation of women ages 45 and older [29]. The sample size was small, but we had a fairly high overall coverage of the population, given that our study sample represented 50.5% of women aged 20–69 in the four recruitment communities. Furthermore, we assessed time-inappropriate screening as not having a Pap smear within the previous 3 years, but if we had a larger sample it would have been important to assess screening compliance in situations where a shorter interval is needed, such as the follow-up of an equivocal or borderline abnormality in the cytology result, where it is recommended that women have a repeat cytology within 6 to 12 months [21].

A major strength of this study was the use of a retrospective medical chart review to determine women’s cervical cancer screening history. Given that women tend to over-report their screening history, by obtaining this variable through chart review, we have reduced bias due to measurement error of the outcome variable. As this analysis was part of a larger research program on HPV and cervical cancer screening, we were able to utilize baseline questionnaire data and thus investigate the association with sexual health and reproductive history factors. This study is one of the few studies to be able to analyse the association between these behaviours and Pap screening. Despite the sensitive nature of many of these covariates, we had relatively low levels of missing data (range: 0-8%).

Understanding screening patterns and groups noncompliant with screening is highly relevant to future efforts to reduce the higher cervical cancer incidence and mortality among Inuit women in Nunavik. Although we found screening rates in Nunavik to be comparable to that of the Canadian population, there is cause for concern given the higher incidence of cervical cancer. Further investigation is needed to determine if factors that take place after screening, such as time to treatment, contribute to the differential risk of cervical cancer mortality between these populations.

## Conclusions

Given that older women and women who are not accessing reproductive care have a lower compliance with time-appropriate cervical cancer screening, future research should address potential strategies to increase Pap smear use or other screening technique such as HPV testing [21] among this group. Deployment of the latter test, owing to its higher sensitivity relative to cytology could permit safely extending screening intervals [33,34]. Moreover, it can be performed in self-collected cervicovaginal samples, which would permit increased coverage to women in remote areas or to those who resist attending screening. We intend to evaluate the potential for such practical screening strategies as a means to provide more cost-effective cervical cancer prevention to these disadvantaged communities.

## Competing interests

ELF has served as occasional consultant to companies involved with HPV vaccines (Merck and GSK) or with cervical cancer screening (Roche, Gen-Probe, BD, Ikonisys). The other authors have no competing interest to declare in relation to this study.

## Authors’ contributions

HC conducted the analysis, interpreted the data and drafted the manuscript. PB conceived the study and participated in its design, data collection coordination and data interpretation. FC and ELF contributed to study design and data interpretation. All authors read and approved the final manuscript.

## Pre-publication history

The pre-publication history for this paper can be accessed here:

http://www.biomedcentral.com/1471-2458/13/438/prepub
